# 1-(4-Bromo-3,5,5,6,8,8-hexa­methyl-5,6,7,8-tetra­hydro­naphthalen-2-yl)ethan-1-one: a precursor for phase-I metabolite of AHTN

**DOI:** 10.1107/S1600536813006934

**Published:** 2013-03-16

**Authors:** Paul Kuhlich, Franziska Emmerling, Werner Kraus, Irene Nehls, Christian Piechotta

**Affiliations:** a BAM Federal Institute for Materials Research and Testing, Richard-Willstätter-Strasse 11, D-12489 Berlin-Adlershof, Germany

## Abstract

The title compound, C_18_H_25_BrO, crystallized as a racemate with four independent mol­ecules in the asymmetric unit. In the crystal, three of these four mol­ecules are linked *via* C—Br⋯Br—C halogen bonds [Br⋯Br = 3.662 (2) and 3.652 (2) Å], forming dimers.

## Related literature

For the crystal structure of the starting material, see: De Ridder *et al.* (1990 ▶). For the next synthesis step for the title compound (aryl halide to phenol), see: Tlili *et al.* (2009 ▶). For possible abiotic and biotic transformation products of AHTN and HHCB, see: Biselli *et al.* (2004 ▶); Martin *et al.* (2007 ▶); Kuhlich *et al.* (2010 ▶); Kuhlich, Emmerling *et al.* (2011 ▶); Kuhlich, Göstl *et al.* (2011 ▶); Faust *et al.* (2011 ▶). For model biotic conversion by liver microsomes, see: Esslinger *et al.* (2011 ▶). For environmental occurrence of AHTN, see: Heberer (2003 ▶). For information on type I and type II halogen inter­actions, see: Pedireddi *et al.* (1994 ▶). For puckering parameters, see: Cremer & Pople (1975 ▶).
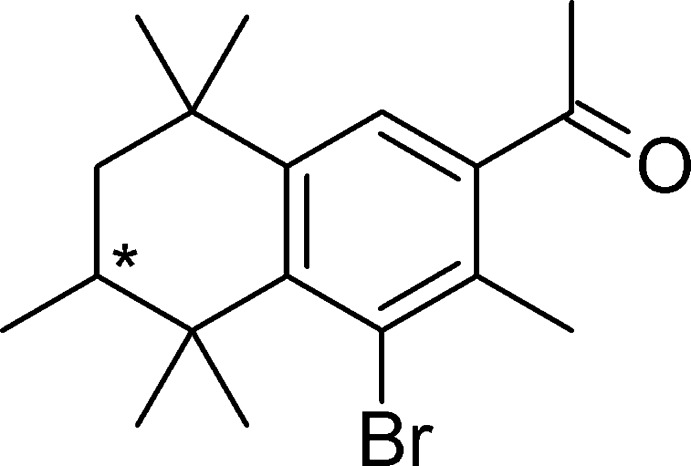


## Experimental

### 

#### Crystal data

C_18_H_25_BrO
*M* *_r_* = 337.28
Monoclinic, 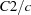

*a* = 35.007 (8) Å
*b* = 19.760 (5) Å
*c* = 24.826 (10) Å
β = 127.681 (6)°
*V* = 13591 (7) Å^3^
*Z* = 32
Mo *K*α radiation
μ = 2.42 mm^−1^
*T* = 293 K
0.25 × 0.18 × 0.1 mm

#### Data collection

Bruker APEX CCD area-detector diffractometer
Absorption correction: multi-scan (*SADABS*; Bruker, 2001 ▶) *T*_min_ = 0.85, *T*_max_ = 0.96
35989 measured reflections
11040 independent reflections
5702 reflections with *I* > 2σ(*I*)
*R*_int_ = 0.051

#### Refinement

*R*[*F* ^2^ > 2σ(*F* ^2^)] = 0.063
*wR*(*F* ^2^) = 0.147
*S* = 1.17
11040 reflections
737 parameters
H-atom parameters constrained
Δρ_max_ = 0.87 e Å^−3^
Δρ_min_ = −0.59 e Å^−3^

### 

Data collection: *SMART* (Bruker, 2001 ▶); cell refinement: *SAINT* (Bruker, 2001 ▶); data reduction: *SAINT*; program(s) used to solve structure: *SHELXS97* (Sheldrick, 2008 ▶); program(s) used to refine structure: *SHELXL97* (Sheldrick, 2008 ▶); molecular graphics: *SHELXTL* (Sheldrick, 2008 ▶) and *ORTEPIII* (Burnett & Johnson, 1996 ▶); software used to prepare material for publication: *SHELXTL*.

## Supplementary Material

Crystal structure: contains datablock(s) I, global. DOI: 10.1107/S1600536813006934/bt6897sup1.cif

Structure factors: contains datablock(s) I. DOI: 10.1107/S1600536813006934/bt6897Isup2.hkl

Supplementary material file. DOI: 10.1107/S1600536813006934/bt6897Isup3.cml

Additional supplementary materials:  crystallographic information; 3D view; checkCIF report

## Figures and Tables

**Fig. 1.**
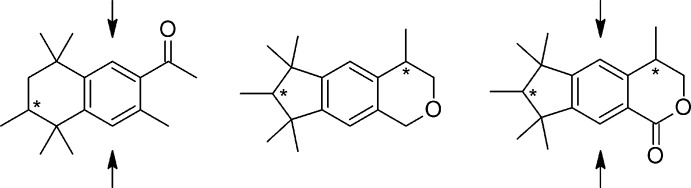
: Line draws of polycyclic musks AHTN (left) and HHCB (middle) as well as line draw of HHCB-Lactone (right). Arrows indicate controversial positions for further hydroxylation.

**Fig. 2.**
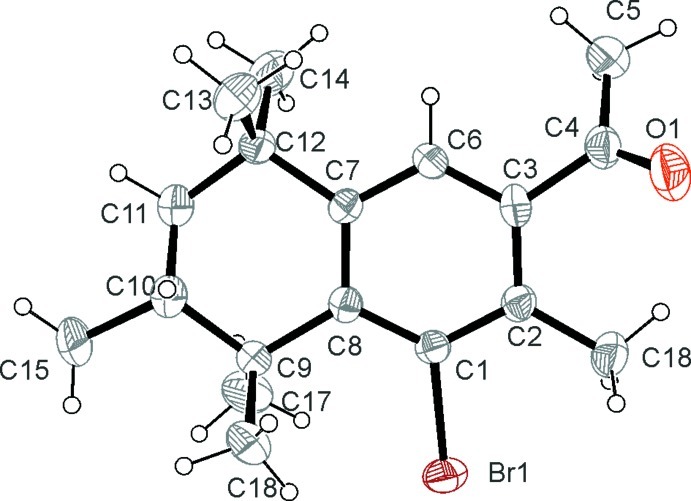
: ORTEP representation of the title compound (molecule A) with atomic labeling shown with 30% probability displacement ellipsoids.

**Fig. 3.**
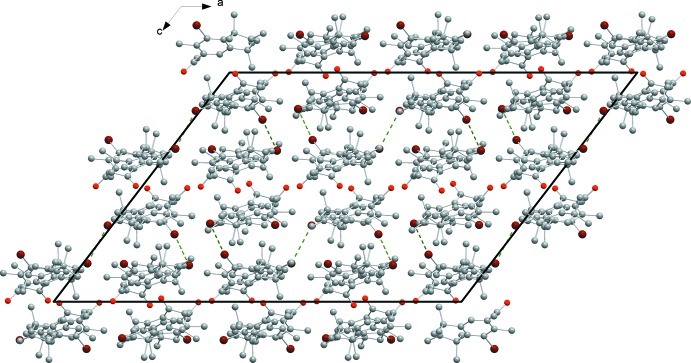
: View of the unit cell of the title compound along [010] showing the C—Br···Br—C halogen bonds between adjacent molecules drawn as dashed green lines.

## supplementary crystallographic information

### Comment

The title compound is the product of an electrophilic aromatic substitution by dibromoisocyanuric acid with anhydrous sulfuric acid. This aryl bromide shall serve as precursor for the introduction of an aromatic hydroxyl group in *meta* position towards the keto-function of AHTN (Tlili *et al.*, 2009).

6-acetyl-1,1,2,4,4,7-hexamethyltetraline (AHTN) and 1,3,4,6,7,8-Hexahydro-4,6,6,7,8,8-hexamethylcyclopenta-γ-2-benzopyran (HHCB) are the predominant representatives of polycyclic musks (Fig. 1). They are widely used fragrances in cosmetics, perfumes, and cleaning products. Produced in ton-scale, they can be found in many different environmental compartments, *e.g.* surface water (Heberer, 2003). Recently, our group investigated anthropogenic transformation pathways of AHTN and HHCB (Kuhlich, Göstl *et al.*, 2011) by disinfection and reported crystal structures of other possible abiotic transformations of AHTN like AHTN-COOH (Kuhlich *et al.*, 2010), AHTN-OH (Faust *et al.*, 2011), and AHTN-COOMe (Kuhlich, Emmerling *et al.*, 2011). The solid state structure of AHTN itself was reported by De Ridder *et al.* (1990).

In literature contrary opinions exist about possible positions for hydroxylation of AHTN. Biselli *et al.* (2004) suggested aromatic hydroxylation for the structure related HHCB-Lactone, whereas Martin *et al.* (2007) did not find hints for their existence by examining the influence of two mitosporic aquatic fungi towards AHTN (supplementary part of that paper). However, Biselli *et al.* did not mention if aromatic hydroxylation occurs *via* an abiotic or biotic pathway. The discussed positions of aromatic hydroxylation are indicated using arrows in Fig. 1.

Our group examined the microsomal conversion of AHTN by human liver microsomes and found three possible metabolites. A various number of conceivable metabolites were synthesized to get references in retention times and fragmentation patterns in comparison to the achieved compounds from incubation using human liver microsomes. A modified protocol for microsomal conversion using rat liver can be found in the literature (Esslinger *et al.*, 2011).

The compound crystallizes in the monoclinic space group *C*2/*c*. The molecular structure of the compound and the atom-labeling scheme are displayed in Fig. 2. Four independent molecules can be found in the asymmetric unit. Two of them (molecule A and D) show a slight disorder which can be deduced from the shape of the ellipsoides. A general puckering analysis of the non-aromatic ring according to Cremer and Pople (Cremer & Pople, 1975) led to a half-chair conformation. The rings (molecule A: C7—C12, molecule B: C25—C30, molecule C: C43—C48, molecule D: C61—C66. For molecule A, see Fig. 2, molecules B—D not shown in detail) have a puckering amplitude (Q) of 0.431 (7) Å (molecule A), 0.447 (7) Å (molecule B), 0.354 (9) Å (molecule C), and 0.301 (10) Å (molecule D), respectively. The maximum deviation from planarity is 0.282 (7) for C10 (molecule A), 0.288 (7) Å for C28 (molecule B), 0.238 (10) Å for C47 (molecule C), and -0.199 (11) Å for C64 (molecule D), respectively.

Three of the four bromine atoms form C—Br···Br—C halogen bonds to adjacent molecules along the [0 0 1] direction (see dashed green bonds in Fig. 3). Between two of the bromine atoms (Br2, Br3) a type I halogen interactions can be observed (Pedireddi *et al.*, 1994). These halogen···halogen contacts C—*X*···*X*—C are defined as type I if the C—*X*···*X* angle α1 is equal or nearly equal to the *X*···*X*—C angle α2 and close to 180° (α1,2=140.65°, d_Br—Br_=3.662 (2) A). Type I contacts arise as a result of close packing about an inversion center. The other bromine atoms (Br1, Br4) are engaged in type II halogen···halogen contacts, where α1 is equal or nearly equal to 180° and α2 is equal or nearly equal to 90° (α1=168.04°, α2= 85.54°, d_Br—Br_=3.652 (2) A).

### Experimental

A solution of AHTN (5.17 g, 20 mmol) in 20 ml chloroform was cooled to 273 K in a 50 ml brown and round bottom flask. Over a time period of 72 h, each day 20 mg of dibromoisocyanuric acid and 2 ml of anhydrous sulfuric acid were added. The reaction mixture was quenched by carefully adding it to 50 ml of brine. The mixture was extracted with dichloromethane (3 *x* 30 ml). The organic extracts were combined, dried over anhydrous sodium sulfate and filtered. After evaporation of the solvent under vacuum, the residue was cleaned by column chromatography (silica gel; dichloromethane). The brown oil dissolved in methanol gave brown crystals overnight (0.8 g, 2.4 mmol, yield: 12%).

^1^H-NMR (500 MHz; CD_3_OD; TMS): δ [p.p.m.] = 7.53 (1*H*, s), 2.55 (3*H*, s), 2.43 (3*H*, s), 1.86 (1*H*, ddq, *J*_H,H'_=2.3 Hz, *J*_H,H''_=13.4 Hz, *J*_H,Me_=6.7 Hz), 1.66 (1*H*, dd, ^2^J=13.5 Hz, ^3^J=13.4 Hz), 1.64 (3*H*, s), 1.44 (3*H*, s), 1.37 (1*H*, dd, ^2^J=13. Hz, ^3^J=2.3 Hz), 1.36 (3*H*, s), 1.26 (3*H*, s), and 1.05 (3*H*, d, J=6.7 Hz); ^13^C-NMR (125 MHz, CD_3_OD, TMS): δ [p.p.m.] = 206.0, 149.3, 147.9, 141.1, 136.0, 130.6, 127.2, 43.7, 41.6, 39.4, 36.9, 33.8, 32.3, 30.8, 27.7, 22.3, 19.2, and 17.7. (+)-ESI/MS: 337.2 (62) [*M*(^79^Br)+H^+^], 339.2 (63) [*M*(^81^Br)+H^+^], 359.2 (100) [*M*(^79^Br)+Na^+^], 361.2 (95) [*M*(^81^Br)+Na^+^].

### Refinement

All H-atoms were positioned geometrically and refined using a riding model with d(C—H) = 0.93 Å, *U*_iso_=1.2*U*_eq_ (C) for aromatic 0.98 Å, *U*_iso_ = 1.2*U*_eq_ (C) for CH, and 0.97 Å, *U*_iso_ = 1.2*U*_eq_ (C) for CH_2_.

### Crystal data

**Table:**

C_18_H_25_BrO	*F*(000) = 5631
*M**_r_* = 337.28	*D*_x_ = 1.319 Mg m^−^^3^
Monoclinic, *C*2/*c*	Mo *K*α radiation, λ = 0.71073 Å
Hall symbol: -C 2yc	Cell parameters from 5584 reflections
*a* = 35.007 (8) Å	θ = 2.3–25.7°
*b* = 19.760 (5) Å	µ = 2.42 mm^−^^1^
*c* = 24.826 (10) Å	*T* = 293 K
β = 127.681 (6)°	Block, light brown
*V* = 13591 (7) Å^3^	0.25 × 0.18 × 0.1 mm
*Z* = 32	

### Data collection

**Table:**

Bruker APEX CCD area-detector diffractometer	11040 independent reflections
Radiation source: fine-focus sealed tube	5702 reflections with *I* > 2σ(*I*)
Graphite monochromator	*R*_int_ = 0.051
ω/2θ scans	θ_max_ = 26.3°, θ_min_ = 1.3°
Absorption correction: multi-scan (*SADABS*; Bruker, 2001)	*h* = −42→40
*T*_min_ = 0.85, *T*_max_ = 0.96	*k* = −24→22
35989 measured reflections	*l* = −26→29

### Refinement

**Table:**

Refinement on *F*^2^	Primary atom site location: structure-invariant direct methods
Least-squares matrix: full	Secondary atom site location: difference Fourier map
*R*[*F*^2^ > 2σ(*F*^2^)] = 0.063	Hydrogen site location: inferred from neighbouring sites
*wR*(*F*^2^) = 0.147	H-atom parameters constrained
*S* = 1.17	*w* = 1/[σ^2^(*F*_o_^2^) + (0.0553*P*)^2^] where *P* = (*F*_o_^2^ + 2*F*_c_^2^)/3
11040 reflections	(Δ/σ)_max_ = 0.003
737 parameters	Δρ_max_ = 0.87 e Å^−^^3^
0 restraints	Δρ_min_ = −0.59 e Å^−^^3^

### Special details

**Table:**

Geometry. All e.s.d.'s (except the e.s.d. in the dihedral angle between two l.s. planes) are estimated using the full covariance matrix. The cell e.s.d.'s are taken into account individually in the estimation of e.s.d.'s in distances, angles and torsion angles; correlations between e.s.d.'s in cell parameters are only used when they are defined by crystal symmetry. An approximate (isotropic) treatment of cell e.s.d.'s is used for estimating e.s.d.'s involving l.s. planes.
Refinement. Refinement of *F*^2^ against ALL reflections. The weighted *R*-factor *wR* and goodness of fit *S* are based on *F*^2^, conventional *R*-factors *R* are based on *F*, with *F* set to zero for negative *F*^2^. The threshold expression of *F*^2^ > σ(*F*^2^) is used only for calculating *R*-factors(gt) *etc*. and is not relevant to the choice of reflections for refinement. *R*-factors based on *F*^2^ are statistically about twice as large as those based on *F*, and *R*- factors based on ALL data will be even larger.

### Fractional atomic coordinates and isotropic or equivalent isotropic displacement parameters (Å^2^)

**Table:**

	*x*	*y*	*z*	*U*_iso_*/*U*_eq_	
Br1	0.26264 (2)	0.32462 (3)	0.34035 (3)	0.0744 (2)	
O1	0.24193 (16)	0.4797 (2)	0.5130 (2)	0.0896 (13)	
C1	0.22543 (16)	0.3259 (2)	0.3744 (2)	0.0431 (12)	
C2	0.22961 (17)	0.3870 (2)	0.4062 (2)	0.0489 (13)	
C3	0.20205 (18)	0.3923 (2)	0.4304 (2)	0.0501 (13)	
C4	0.2038 (2)	0.4534 (3)	0.4680 (3)	0.0594 (15)	
C5	0.1570 (2)	0.4817 (3)	0.4484 (3)	0.0786 (17)	
H5A	0.1634	0.5211	0.4753	0.118*	
H5B	0.1410	0.4482	0.4563	0.118*	
H5C	0.1367	0.4937	0.4010	0.118*	
C6	0.17131 (17)	0.3407 (2)	0.4178 (2)	0.0492 (13)	
H6	0.1516	0.3469	0.4307	0.059*	
C7	0.16781 (16)	0.2798 (2)	0.3871 (2)	0.0427 (12)	
C8	0.19698 (16)	0.2702 (2)	0.3657 (2)	0.0423 (12)	
C9	0.19702 (18)	0.2019 (2)	0.3353 (3)	0.0525 (13)	
C10	0.1702 (2)	0.1468 (3)	0.3452 (3)	0.0808 (13)	
H10	0.1919	0.1359	0.3938	0.097*	
C11	0.1271 (2)	0.1687 (3)	0.3327 (3)	0.0808 (13)	
H11A	0.1126	0.1304	0.3385	0.097*	
H11B	0.1049	0.1828	0.2855	0.097*	
C12	0.13189 (18)	0.2268 (2)	0.3774 (3)	0.0548 (14)	
C13	0.08205 (19)	0.2593 (3)	0.3399 (3)	0.0908 (19)	
H13A	0.0590	0.2256	0.3309	0.136*	
H13B	0.0725	0.2782	0.2977	0.136*	
H13C	0.0833	0.2945	0.3676	0.136*	
C14	0.1480 (2)	0.2015 (3)	0.4462 (3)	0.0804 (18)	
H14A	0.1782	0.1782	0.4688	0.121*	
H14B	0.1241	0.1711	0.4398	0.121*	
H14C	0.1518	0.2393	0.4734	0.121*	
C15	0.1632 (2)	0.0792 (3)	0.3083 (3)	0.097 (2)	
H15A	0.1533	0.0445	0.3246	0.145*	
H15B	0.1931	0.0664	0.3173	0.145*	
H15C	0.1388	0.0849	0.2602	0.145*	
C16	0.2496 (2)	0.1750 (3)	0.3752 (3)	0.0780 (17)	
H16A	0.2661	0.1805	0.4232	0.117*	
H16B	0.2662	0.2000	0.3620	0.117*	
H16C	0.2489	0.1279	0.3652	0.117*	
C17	0.1733 (2)	0.2107 (3)	0.2584 (2)	0.0815 (18)	
H17A	0.1392	0.2171	0.2332	0.122*	
H17B	0.1792	0.1710	0.2422	0.122*	
H17C	0.1870	0.2495	0.2526	0.122*	
C18	0.26116 (19)	0.4443 (2)	0.4136 (3)	0.0702 (16)	
H18A	0.2942	0.4297	0.4413	0.105*	
H18B	0.2581	0.4823	0.4348	0.105*	
H18C	0.2513	0.4573	0.3695	0.105*	
Br2	−0.00945 (2)	0.07436 (3)	0.16887 (4)	0.0887 (3)	
O2	0.01668 (15)	0.2345 (2)	0.0076 (2)	0.0880 (13)	
C19	0.02661 (16)	0.0735 (3)	0.1333 (2)	0.0473 (13)	
C20	0.02763 (17)	0.1362 (2)	0.1082 (2)	0.0492 (13)	
C21	0.05389 (16)	0.1409 (2)	0.0827 (2)	0.0460 (12)	
C22	0.0542 (2)	0.2032 (3)	0.0481 (3)	0.0576 (14)	
C23	0.10048 (19)	0.2264 (3)	0.0637 (3)	0.0758 (17)	
H23A	0.0954	0.2684	0.0407	0.114*	
H23B	0.1119	0.1929	0.0487	0.114*	
H23C	0.1240	0.2329	0.1119	0.114*	
C24	0.07961 (17)	0.0851 (2)	0.0878 (2)	0.0501 (13)	
H24	0.0987	0.0895	0.0737	0.060*	
C25	0.07861 (16)	0.0228 (2)	0.1127 (2)	0.0461 (12)	
C26	0.04974 (16)	0.0138 (2)	0.1343 (2)	0.0441 (12)	
C27	0.04296 (18)	−0.0560 (2)	0.1557 (3)	0.0558 (14)	
C28	0.0659 (2)	−0.1123 (3)	0.1402 (3)	0.0864 (19)	
H28	0.0438	−0.1187	0.0908	0.104*	
C29	0.1112 (2)	−0.0940 (3)	0.1559 (3)	0.0882 (19)	
H29A	0.1236	−0.1329	0.1472	0.106*	
H29B	0.1336	−0.0843	0.2042	0.106*	
C30	0.11107 (19)	−0.0330 (3)	0.1170 (3)	0.0573 (14)	
C31	0.0938 (2)	−0.0541 (3)	0.0462 (3)	0.093 (2)	
H31A	0.0612	−0.0704	0.0204	0.139*	
H31B	0.1143	−0.0893	0.0503	0.139*	
H31C	0.0950	−0.0159	0.0235	0.139*	
C32	0.16306 (19)	−0.0080 (3)	0.1567 (3)	0.0851 (18)	
H32A	0.1646	0.0272	0.1314	0.128*	
H32B	0.1835	−0.0449	0.1635	0.128*	
H32C	0.1738	0.0094	0.2000	0.128*	
C33	0.0687 (3)	−0.1818 (3)	0.1710 (4)	0.113 (2)	
H33A	0.0758	−0.2163	0.1511	0.170*	
H33B	0.0383	−0.1915	0.1617	0.170*	
H33C	0.0937	−0.1808	0.2193	0.170*	
C34	−0.0113 (2)	−0.0748 (3)	0.1114 (3)	0.0851 (18)	
H34A	−0.0267	−0.0493	0.1261	0.128*	
H34B	−0.0145	−0.1223	0.1159	0.128*	
H34C	−0.0262	−0.0645	0.0646	0.128*	
C35	0.0651 (2)	−0.0566 (3)	0.2324 (3)	0.0819 (18)	
H35A	0.0996	−0.0567	0.2597	0.123*	
H35B	0.0545	−0.0964	0.2420	0.123*	
H35C	0.0547	−0.0171	0.2427	0.123*	
C36	0.00293 (19)	0.1976 (2)	0.1111 (3)	0.0702 (16)	
H36A	−0.0314	0.1908	0.0814	0.105*	
H36B	0.0107	0.2370	0.0968	0.105*	
H36C	0.0139	0.2039	0.1569	0.105*	
Br3	0.39963 (3)	0.15741 (3)	0.16812 (4)	0.1016 (3)	
O3	0.35630 (15)	0.3856 (2)	0.0044 (2)	0.0942 (14)	
C37	0.35939 (18)	0.2373 (2)	0.1354 (2)	0.0550 (14)	
C38	0.37308 (18)	0.2858 (3)	0.1100 (3)	0.0551 (14)	
C39	0.34572 (18)	0.3446 (3)	0.0847 (2)	0.0485 (13)	
C40	0.35430 (18)	0.3989 (3)	0.0503 (3)	0.0614 (15)	
C41	0.3602 (2)	0.4699 (3)	0.0747 (3)	0.0734 (16)	
H41A	0.3669	0.4989	0.0504	0.110*	
H41B	0.3865	0.4721	0.1225	0.110*	
H41C	0.3310	0.4845	0.0669	0.110*	
C42	0.30823 (18)	0.3527 (2)	0.0888 (2)	0.0508 (13)	
H42	0.2911	0.3932	0.0732	0.061*	
C43	0.29456 (16)	0.3039 (2)	0.1147 (2)	0.0437 (12)	
C44	0.31989 (17)	0.2424 (2)	0.1380 (2)	0.0451 (12)	
C45	0.30483 (18)	0.1835 (2)	0.1637 (3)	0.0531 (14)	
C46	0.2575 (3)	0.2015 (3)	0.1527 (4)	0.103 (2)	
H46	0.2330	0.1924	0.1041	0.123*	
C47	0.2495 (3)	0.2685 (4)	0.1572 (4)	0.120 (3)	
H47A	0.2723	0.2815	0.2047	0.145*	
H47B	0.2175	0.2719	0.1454	0.145*	
C48	0.25257 (18)	0.3207 (3)	0.1162 (3)	0.0565 (14)	
C49	0.2611 (2)	0.3901 (3)	0.1510 (3)	0.099 (2)	
H49A	0.2368	0.3976	0.1569	0.148*	
H49B	0.2595	0.4254	0.1230	0.148*	
H49C	0.2924	0.3905	0.1946	0.148*	
C50	0.20561 (19)	0.3266 (3)	0.0438 (3)	0.095 (2)	
H50A	0.1995	0.2849	0.0199	0.143*	
H50B	0.2083	0.3628	0.0205	0.143*	
H50C	0.1794	0.3359	0.0455	0.143*	
C51	0.2432 (2)	0.1519 (3)	0.1852 (4)	0.118 (3)	
H51A	0.2638	0.1586	0.2336	0.177*	
H51B	0.2467	0.1063	0.1754	0.177*	
H51C	0.2103	0.1596	0.1670	0.177*	
C52	0.2933 (2)	0.1197 (3)	0.1215 (3)	0.0836 (18)	
H52A	0.3223	0.1027	0.1303	0.125*	
H52B	0.2703	0.1303	0.0740	0.125*	
H52C	0.2801	0.0860	0.1337	0.125*	
C53	0.3453 (2)	0.1696 (3)	0.2399 (3)	0.090 (2)	
H53A	0.3389	0.1276	0.2526	0.135*	
H53B	0.3463	0.2057	0.2666	0.135*	
H53C	0.3757	0.1668	0.2478	0.135*	
C54	0.41666 (19)	0.2769 (3)	0.1108 (3)	0.089 (2)	
H54A	0.4219	0.3177	0.0952	0.134*	
H54B	0.4109	0.2401	0.0814	0.134*	
H54C	0.4447	0.2672	0.1564	0.134*	
Br4	0.16887 (3)	0.43574 (4)	0.20596 (4)	0.1130 (3)	
O4	0.11442 (16)	0.6364 (2)	0.0060 (2)	0.1014 (15)	
C55	0.11951 (18)	0.5017 (2)	0.1502 (2)	0.0537 (14)	
C56	0.13121 (18)	0.5477 (3)	0.1196 (3)	0.0525 (13)	
C57	0.09929 (18)	0.6003 (2)	0.0831 (2)	0.0466 (13)	
C58	0.10742 (18)	0.6520 (3)	0.0466 (3)	0.0613 (15)	
C59	0.1069 (2)	0.7255 (3)	0.0625 (3)	0.0816 (18)	
H59A	0.1108	0.7534	0.0346	0.122*	
H59B	0.1328	0.7340	0.1096	0.122*	
H59C	0.0767	0.7359	0.0535	0.122*	
C60	0.05860 (18)	0.6056 (2)	0.0795 (2)	0.0493 (13)	
H60	0.0386	0.6429	0.0571	0.059*	
C61	0.04530 (16)	0.5584 (2)	0.1075 (2)	0.0430 (12)	
C62	0.07616 (18)	0.5032 (2)	0.1437 (2)	0.0468 (13)	
C63	0.0624 (2)	0.4471 (3)	0.1724 (3)	0.0630 (15)	
C64	0.0108 (3)	0.4575 (4)	0.1475 (5)	0.134 (2)	
H64	−0.0064	0.4433	0.1001	0.161*	
C65	−0.0071 (3)	0.5184 (4)	0.1369 (4)	0.134 (2)	
H65A	0.0065	0.5377	0.1812	0.161*	
H65B	−0.0415	0.5132	0.1137	0.161*	
C66	−0.00145 (18)	0.5707 (3)	0.0977 (3)	0.0573 (14)	
C67	−0.0442 (2)	0.5717 (3)	0.0231 (3)	0.101 (2)	
H67A	−0.0387	0.6045	−0.0001	0.152*	
H67B	−0.0727	0.5835	0.0182	0.152*	
H67C	−0.0482	0.5277	0.0039	0.152*	
C68	0.0001 (2)	0.6411 (3)	0.1268 (3)	0.098 (2)	
H68A	0.0001	0.6760	0.0999	0.146*	
H68B	0.0288	0.6446	0.1730	0.146*	
H68C	−0.0278	0.6462	0.1255	0.146*	
C69	−0.0090 (3)	0.4044 (4)	0.1697 (4)	0.178 (4)	
H69A	−0.0098	0.4230	0.2047	0.267*	
H69B	0.0114	0.3652	0.1869	0.267*	
H69C	−0.0411	0.3918	0.1314	0.267*	
C70	0.0973 (2)	0.4504 (3)	0.2513 (3)	0.096 (2)	
H70A	0.0885	0.4164	0.2695	0.144*	
H70B	0.0953	0.4943	0.2660	0.144*	
H70C	0.1298	0.4426	0.2672	0.144*	
C71	0.0640 (3)	0.3773 (3)	0.1472 (3)	0.113 (2)	
H71A	0.0361	0.3717	0.1007	0.170*	
H71B	0.0641	0.3430	0.1747	0.170*	
H71C	0.0927	0.3735	0.1505	0.170*	
C72	0.17772 (19)	0.5424 (3)	0.1270 (3)	0.0868 (19)	
H72A	0.1771	0.5019	0.1051	0.130*	
H72B	0.2049	0.5411	0.1744	0.130*	
H72C	0.1805	0.5811	0.1061	0.130*	

### Atomic displacement parameters (Å^2^)

**Table:**

	*U*^11^	*U*^22^	*U*^33^	*U*^12^	*U*^13^	*U*^23^
Br1	0.0794 (5)	0.0681 (4)	0.1107 (5)	0.0019 (3)	0.0760 (4)	0.0058 (4)
O1	0.095 (3)	0.072 (3)	0.087 (3)	−0.017 (3)	0.048 (3)	−0.029 (2)
C1	0.043 (3)	0.041 (3)	0.048 (3)	0.006 (3)	0.029 (3)	0.008 (3)
C2	0.049 (3)	0.039 (3)	0.058 (3)	0.003 (3)	0.033 (3)	0.007 (3)
C3	0.057 (4)	0.038 (3)	0.046 (3)	−0.006 (3)	0.026 (3)	−0.006 (3)
C4	0.074 (5)	0.044 (4)	0.065 (4)	−0.006 (3)	0.045 (4)	−0.003 (3)
C5	0.096 (5)	0.064 (4)	0.099 (5)	−0.002 (4)	0.072 (4)	−0.009 (3)
C6	0.052 (3)	0.046 (3)	0.055 (3)	0.001 (3)	0.035 (3)	−0.007 (3)
C7	0.041 (3)	0.043 (3)	0.043 (3)	−0.001 (3)	0.025 (3)	0.003 (2)
C8	0.040 (3)	0.039 (3)	0.041 (3)	0.004 (3)	0.021 (3)	0.006 (2)
C9	0.054 (4)	0.046 (3)	0.065 (4)	−0.003 (3)	0.041 (3)	−0.007 (3)
C10	0.095 (4)	0.059 (3)	0.116 (3)	−0.024 (3)	0.079 (3)	−0.027 (3)
C11	0.095 (4)	0.059 (3)	0.116 (3)	−0.024 (3)	0.079 (3)	−0.027 (3)
C12	0.051 (4)	0.046 (3)	0.077 (4)	0.004 (3)	0.044 (3)	0.003 (3)
C13	0.066 (5)	0.089 (5)	0.121 (5)	−0.012 (4)	0.058 (4)	−0.005 (4)
C14	0.097 (5)	0.075 (4)	0.090 (5)	−0.006 (4)	0.067 (4)	0.011 (4)
C15	0.135 (6)	0.057 (4)	0.138 (6)	−0.032 (4)	0.104 (5)	−0.044 (4)
C16	0.084 (5)	0.057 (4)	0.105 (5)	0.015 (3)	0.064 (4)	0.003 (3)
C17	0.089 (5)	0.102 (5)	0.059 (4)	0.006 (4)	0.049 (4)	−0.007 (3)
C18	0.072 (4)	0.049 (4)	0.091 (4)	−0.009 (3)	0.051 (4)	−0.002 (3)
Br2	0.1090 (6)	0.0835 (5)	0.1288 (6)	0.0111 (4)	0.1009 (5)	0.0116 (4)
O2	0.078 (3)	0.072 (3)	0.102 (3)	0.021 (2)	0.049 (3)	0.036 (2)
C19	0.045 (3)	0.053 (4)	0.046 (3)	−0.001 (3)	0.030 (3)	−0.002 (3)
C20	0.050 (3)	0.041 (3)	0.054 (3)	0.004 (3)	0.031 (3)	−0.004 (3)
C21	0.046 (3)	0.041 (3)	0.049 (3)	0.002 (3)	0.027 (3)	0.001 (3)
C22	0.077 (5)	0.043 (3)	0.060 (4)	0.009 (3)	0.046 (4)	0.003 (3)
C23	0.079 (4)	0.074 (4)	0.093 (4)	0.002 (3)	0.062 (4)	0.023 (3)
C24	0.054 (4)	0.044 (3)	0.062 (3)	0.004 (3)	0.040 (3)	0.005 (3)
C25	0.044 (3)	0.044 (3)	0.048 (3)	0.006 (3)	0.027 (3)	−0.001 (3)
C26	0.045 (3)	0.045 (3)	0.041 (3)	0.000 (3)	0.026 (3)	−0.004 (2)
C27	0.058 (4)	0.046 (3)	0.062 (4)	−0.002 (3)	0.036 (3)	0.007 (3)
C28	0.096 (5)	0.057 (4)	0.118 (5)	0.014 (4)	0.071 (5)	0.019 (4)
C29	0.099 (6)	0.058 (4)	0.111 (5)	0.029 (4)	0.066 (5)	0.017 (4)
C30	0.061 (4)	0.050 (4)	0.063 (4)	0.007 (3)	0.039 (3)	0.007 (3)
C31	0.113 (5)	0.093 (5)	0.082 (5)	0.023 (4)	0.065 (4)	−0.005 (4)
C32	0.067 (4)	0.086 (5)	0.102 (5)	0.015 (4)	0.052 (4)	−0.001 (4)
C33	0.164 (7)	0.049 (4)	0.162 (7)	0.017 (4)	0.117 (6)	0.029 (4)
C34	0.075 (5)	0.068 (4)	0.094 (5)	−0.019 (4)	0.042 (4)	0.003 (3)
C35	0.092 (5)	0.089 (5)	0.060 (4)	−0.011 (4)	0.045 (4)	0.015 (3)
C36	0.086 (4)	0.054 (4)	0.095 (4)	0.011 (3)	0.067 (4)	0.004 (3)
Br3	0.1022 (6)	0.0822 (5)	0.1513 (7)	0.0428 (4)	0.0933 (5)	0.0474 (5)
O3	0.136 (4)	0.092 (3)	0.099 (3)	−0.023 (3)	0.095 (3)	−0.007 (3)
C37	0.052 (4)	0.048 (3)	0.060 (4)	0.009 (3)	0.032 (3)	0.007 (3)
C38	0.052 (4)	0.056 (4)	0.068 (4)	0.000 (3)	0.042 (3)	−0.001 (3)
C39	0.048 (3)	0.050 (3)	0.053 (3)	−0.004 (3)	0.034 (3)	−0.003 (3)
C40	0.056 (4)	0.069 (4)	0.067 (4)	−0.004 (3)	0.042 (3)	0.003 (3)
C41	0.086 (4)	0.063 (4)	0.081 (4)	−0.009 (3)	0.055 (4)	0.008 (3)
C42	0.059 (4)	0.043 (3)	0.051 (3)	0.004 (3)	0.034 (3)	0.001 (3)
C43	0.041 (3)	0.047 (3)	0.041 (3)	0.000 (3)	0.024 (3)	0.001 (3)
C44	0.046 (3)	0.046 (3)	0.044 (3)	−0.006 (3)	0.028 (3)	−0.005 (3)
C45	0.058 (4)	0.046 (3)	0.060 (4)	0.001 (3)	0.038 (3)	0.003 (3)
C46	0.134 (6)	0.061 (5)	0.189 (7)	0.003 (4)	0.137 (6)	0.019 (5)
C47	0.123 (6)	0.119 (7)	0.192 (8)	0.030 (5)	0.133 (6)	0.055 (6)
C48	0.050 (4)	0.061 (4)	0.069 (4)	0.000 (3)	0.041 (3)	0.006 (3)
C49	0.089 (5)	0.110 (6)	0.119 (5)	0.007 (4)	0.075 (5)	−0.025 (4)
C50	0.046 (4)	0.135 (6)	0.077 (5)	0.009 (4)	0.023 (4)	−0.005 (4)
C51	0.124 (6)	0.113 (6)	0.173 (7)	0.000 (5)	0.120 (6)	0.035 (5)
C52	0.109 (5)	0.061 (4)	0.092 (5)	−0.014 (4)	0.067 (4)	−0.002 (3)
C53	0.112 (5)	0.084 (5)	0.069 (4)	−0.012 (4)	0.053 (4)	0.017 (4)
C54	0.077 (4)	0.088 (5)	0.133 (5)	0.012 (4)	0.081 (4)	0.022 (4)
Br4	0.1032 (6)	0.1114 (6)	0.1450 (7)	0.0567 (5)	0.0864 (6)	0.0657 (5)
O4	0.165 (4)	0.083 (3)	0.128 (4)	−0.016 (3)	0.126 (4)	−0.004 (3)
C55	0.058 (4)	0.042 (3)	0.066 (4)	0.004 (3)	0.041 (3)	0.002 (3)
C56	0.050 (4)	0.052 (4)	0.058 (3)	0.002 (3)	0.034 (3)	−0.003 (3)
C57	0.053 (4)	0.045 (3)	0.051 (3)	−0.005 (3)	0.036 (3)	−0.001 (3)
C58	0.059 (4)	0.063 (4)	0.075 (4)	−0.003 (3)	0.048 (3)	0.002 (3)
C59	0.101 (5)	0.059 (4)	0.111 (5)	−0.005 (4)	0.078 (4)	0.012 (4)
C60	0.055 (4)	0.043 (3)	0.051 (3)	0.007 (3)	0.032 (3)	0.003 (3)
C61	0.044 (3)	0.041 (3)	0.048 (3)	−0.004 (3)	0.030 (3)	−0.002 (3)
C62	0.053 (4)	0.045 (3)	0.043 (3)	−0.002 (3)	0.030 (3)	−0.004 (3)
C63	0.075 (4)	0.060 (4)	0.063 (4)	0.002 (3)	0.047 (4)	0.018 (3)
C64	0.114 (5)	0.117 (5)	0.226 (6)	0.032 (4)	0.132 (5)	0.084 (5)
C65	0.114 (5)	0.117 (5)	0.226 (6)	0.032 (4)	0.132 (5)	0.084 (5)
C66	0.051 (4)	0.055 (4)	0.070 (4)	0.002 (3)	0.039 (3)	0.009 (3)
C67	0.054 (4)	0.125 (6)	0.098 (5)	−0.002 (4)	0.032 (4)	−0.020 (4)
C68	0.079 (5)	0.123 (6)	0.107 (5)	0.015 (4)	0.065 (4)	−0.021 (4)
C69	0.124 (7)	0.196 (9)	0.226 (10)	−0.003 (6)	0.113 (7)	0.108 (8)
C70	0.121 (6)	0.098 (5)	0.079 (5)	−0.001 (4)	0.067 (5)	0.024 (4)
C71	0.177 (7)	0.047 (4)	0.115 (5)	−0.034 (4)	0.089 (6)	−0.002 (4)
C72	0.071 (4)	0.100 (5)	0.108 (5)	0.014 (4)	0.063 (4)	0.018 (4)

### Geometric parameters (Å, º)

**Table:**

Br1—C1	1.939 (4)	Br3—C37	1.933 (5)
O1—C4	1.216 (6)	O3—C40	1.214 (5)
C1—C2	1.400 (6)	C37—C38	1.385 (6)
C1—C8	1.410 (6)	C37—C44	1.426 (6)
C2—C3	1.420 (6)	C38—C39	1.387 (6)
C2—C18	1.512 (6)	C38—C54	1.523 (6)
C3—C6	1.373 (6)	C39—C42	1.386 (6)
C3—C4	1.502 (7)	C39—C40	1.511 (7)
C4—C5	1.503 (7)	C40—C41	1.491 (7)
C5—H5A	0.9600	C41—H41A	0.9600
C5—H5B	0.9600	C41—H41B	0.9600
C5—H5C	0.9600	C41—H41C	0.9600
C6—C7	1.389 (6)	C42—C43	1.397 (6)
C6—H6	0.9300	C42—H42	0.9300
C7—C8	1.423 (6)	C43—C44	1.405 (6)
C7—C12	1.538 (6)	C43—C48	1.528 (6)
C8—C9	1.545 (6)	C44—C45	1.564 (6)
C9—C10	1.552 (6)	C45—C52	1.528 (6)
C9—C16	1.555 (6)	C45—C53	1.544 (7)
C9—C17	1.557 (6)	C45—C46	1.549 (7)
C10—C11	1.410 (7)	C46—C47	1.371 (8)
C10—C15	1.552 (7)	C46—C51	1.534 (7)
C10—H10	0.9800	C46—H46	0.9800
C11—C12	1.532 (6)	C47—C48	1.501 (7)
C11—H11A	0.9700	C47—H47A	0.9700
C11—H11B	0.9700	C47—H47B	0.9700
C12—C14	1.520 (6)	C48—C50	1.528 (7)
C12—C13	1.529 (7)	C48—C49	1.549 (7)
C13—H13A	0.9600	C49—H49A	0.9600
C13—H13B	0.9600	C49—H49B	0.9600
C13—H13C	0.9600	C49—H49C	0.9600
C14—H14A	0.9600	C50—H50A	0.9600
C14—H14B	0.9600	C50—H50B	0.9600
C14—H14C	0.9600	C50—H50C	0.9600
C15—H15A	0.9600	C51—H51A	0.9600
C15—H15B	0.9600	C51—H51B	0.9600
C15—H15C	0.9600	C51—H51C	0.9600
C16—H16A	0.9600	C52—H52A	0.9600
C16—H16B	0.9600	C52—H52B	0.9600
C16—H16C	0.9600	C52—H52C	0.9600
C17—H17A	0.9600	C53—H53A	0.9600
C17—H17B	0.9600	C53—H53B	0.9600
C17—H17C	0.9600	C53—H53C	0.9600
C18—H18A	0.9600	C54—H54A	0.9600
C18—H18B	0.9600	C54—H54B	0.9600
C18—H18C	0.9600	C54—H54C	0.9600
Br2—C19	1.934 (4)	Br4—C55	1.917 (5)
O2—C22	1.225 (6)	O4—C58	1.212 (5)
C19—C20	1.395 (6)	C55—C56	1.396 (6)
C19—C26	1.422 (6)	C55—C62	1.424 (6)
C20—C21	1.403 (6)	C56—C57	1.384 (6)
C20—C36	1.516 (6)	C56—C72	1.527 (6)
C21—C24	1.381 (6)	C57—C60	1.375 (6)
C21—C22	1.505 (6)	C57—C58	1.505 (7)
C22—C23	1.489 (7)	C58—C59	1.509 (7)
C23—H23A	0.9600	C59—H59A	0.9600
C23—H23B	0.9600	C59—H59B	0.9600
C23—H23C	0.9600	C59—H59C	0.9600
C24—C25	1.386 (6)	C60—C61	1.402 (6)
C24—H24	0.9300	C60—H60	0.9300
C25—C26	1.417 (6)	C61—C62	1.407 (6)
C25—C30	1.539 (6)	C61—C66	1.519 (6)
C26—C27	1.546 (6)	C62—C63	1.545 (6)
C27—C34	1.549 (7)	C63—C64	1.523 (8)
C27—C28	1.553 (7)	C63—C71	1.529 (7)
C27—C35	1.556 (6)	C63—C70	1.551 (7)
C28—C29	1.428 (7)	C64—C65	1.307 (8)
C28—C33	1.545 (7)	C64—C69	1.536 (8)
C28—H28	0.9800	C64—H64	0.9800
C29—C30	1.543 (7)	C65—C66	1.515 (7)
C29—H29A	0.9700	C65—H65A	0.9700
C29—H29B	0.9700	C65—H65B	0.9700
C30—C31	1.527 (7)	C66—C67	1.509 (7)
C30—C32	1.528 (7)	C66—C68	1.553 (7)
C31—H31A	0.9600	C67—H67A	0.9600
C31—H31B	0.9600	C67—H67B	0.9600
C31—H31C	0.9600	C67—H67C	0.9600
C32—H32A	0.9600	C68—H68A	0.9600
C32—H32B	0.9600	C68—H68B	0.9600
C32—H32C	0.9600	C68—H68C	0.9600
C33—H33A	0.9600	C69—H69A	0.9600
C33—H33B	0.9600	C69—H69B	0.9600
C33—H33C	0.9600	C69—H69C	0.9600
C34—H34A	0.9600	C70—H70A	0.9600
C34—H34B	0.9600	C70—H70B	0.9600
C34—H34C	0.9600	C70—H70C	0.9600
C35—H35A	0.9600	C71—H71A	0.9600
C35—H35B	0.9600	C71—H71B	0.9600
C35—H35C	0.9600	C71—H71C	0.9600
C36—H36A	0.9600	C72—H72A	0.9600
C36—H36B	0.9600	C72—H72B	0.9600
C36—H36C	0.9600	C72—H72C	0.9600
			
C2—C1—C8	125.6 (4)	C38—C37—C44	126.0 (4)
C2—C1—Br1	112.9 (3)	C38—C37—Br3	112.9 (4)
C8—C1—Br1	121.5 (3)	C44—C37—Br3	121.1 (4)
C1—C2—C3	116.0 (4)	C37—C38—C39	116.8 (4)
C1—C2—C18	122.2 (4)	C37—C38—C54	122.5 (5)
C3—C2—C18	121.9 (4)	C39—C38—C54	120.8 (5)
C6—C3—C2	119.3 (4)	C42—C39—C38	119.1 (4)
C6—C3—C4	117.9 (5)	C42—C39—C40	118.8 (5)
C2—C3—C4	122.8 (5)	C38—C39—C40	122.0 (4)
O1—C4—C3	121.4 (5)	O3—C40—C41	120.8 (5)
O1—C4—C5	120.3 (5)	O3—C40—C39	121.4 (5)
C3—C4—C5	118.3 (5)	C41—C40—C39	117.8 (5)
C4—C5—H5A	109.5	C40—C41—H41A	109.5
C4—C5—H5B	109.5	C40—C41—H41B	109.5
H5A—C5—H5B	109.5	H41A—C41—H41B	109.5
C4—C5—H5C	109.5	C40—C41—H41C	109.5
H5A—C5—H5C	109.5	H41A—C41—H41C	109.5
H5B—C5—H5C	109.5	H41B—C41—H41C	109.5
C3—C6—C7	124.2 (4)	C39—C42—C43	124.1 (5)
C3—C6—H6	117.9	C39—C42—H42	117.9
C7—C6—H6	117.9	C43—C42—H42	117.9
C6—C7—C8	118.6 (4)	C42—C43—C44	118.6 (4)
C6—C7—C12	117.7 (4)	C42—C43—C48	117.8 (4)
C8—C7—C12	123.7 (4)	C44—C43—C48	123.6 (4)
C1—C8—C7	116.0 (4)	C43—C44—C37	115.3 (4)
C1—C8—C9	123.1 (4)	C43—C44—C45	121.7 (4)
C7—C8—C9	120.9 (4)	C37—C44—C45	122.9 (4)
C8—C9—C10	110.7 (4)	C52—C45—C53	110.6 (4)
C8—C9—C16	109.9 (4)	C52—C45—C46	105.3 (5)
C10—C9—C16	104.7 (4)	C53—C45—C46	109.7 (5)
C8—C9—C17	109.9 (4)	C52—C45—C44	110.4 (4)
C10—C9—C17	110.6 (4)	C53—C45—C44	110.3 (4)
C16—C9—C17	110.8 (4)	C46—C45—C44	110.4 (4)
C11—C10—C9	114.8 (5)	C47—C46—C51	115.2 (5)
C11—C10—C15	111.9 (5)	C47—C46—C45	117.2 (5)
C9—C10—C15	113.7 (4)	C51—C46—C45	114.7 (5)
C11—C10—H10	105.1	C47—C46—H46	102.1
C9—C10—H10	105.1	C51—C46—H46	102.1
C15—C10—H10	105.1	C45—C46—H46	102.1
C10—C11—C12	116.2 (5)	C46—C47—C48	120.3 (6)
C10—C11—H11A	108.2	C46—C47—H47A	107.2
C12—C11—H11A	108.2	C48—C47—H47A	107.2
C10—C11—H11B	108.2	C46—C47—H47B	107.2
C12—C11—H11B	108.2	C48—C47—H47B	107.2
H11A—C11—H11B	107.4	H47A—C47—H47B	106.9
C14—C12—C13	108.8 (4)	C47—C48—C50	112.2 (5)
C14—C12—C11	111.7 (4)	C47—C48—C43	109.7 (4)
C13—C12—C11	107.1 (5)	C50—C48—C43	110.4 (4)
C14—C12—C7	110.0 (4)	C47—C48—C49	107.5 (5)
C13—C12—C7	109.2 (4)	C50—C48—C49	106.9 (5)
C11—C12—C7	109.9 (4)	C43—C48—C49	110.1 (4)
C12—C13—H13A	109.5	C48—C49—H49A	109.5
C12—C13—H13B	109.5	C48—C49—H49B	109.5
H13A—C13—H13B	109.5	H49A—C49—H49B	109.5
C12—C13—H13C	109.5	C48—C49—H49C	109.5
H13A—C13—H13C	109.5	H49A—C49—H49C	109.5
H13B—C13—H13C	109.5	H49B—C49—H49C	109.5
C12—C14—H14A	109.5	C48—C50—H50A	109.5
C12—C14—H14B	109.5	C48—C50—H50B	109.5
H14A—C14—H14B	109.5	H50A—C50—H50B	109.5
C12—C14—H14C	109.5	C48—C50—H50C	109.5
H14A—C14—H14C	109.5	H50A—C50—H50C	109.5
H14B—C14—H14C	109.5	H50B—C50—H50C	109.5
C10—C15—H15A	109.5	C46—C51—H51A	109.5
C10—C15—H15B	109.5	C46—C51—H51B	109.5
H15A—C15—H15B	109.5	H51A—C51—H51B	109.5
C10—C15—H15C	109.5	C46—C51—H51C	109.5
H15A—C15—H15C	109.5	H51A—C51—H51C	109.5
H15B—C15—H15C	109.5	H51B—C51—H51C	109.5
C9—C16—H16A	109.5	C45—C52—H52A	109.5
C9—C16—H16B	109.5	C45—C52—H52B	109.5
H16A—C16—H16B	109.5	H52A—C52—H52B	109.5
C9—C16—H16C	109.5	C45—C52—H52C	109.5
H16A—C16—H16C	109.5	H52A—C52—H52C	109.5
H16B—C16—H16C	109.5	H52B—C52—H52C	109.5
C9—C17—H17A	109.5	C45—C53—H53A	109.5
C9—C17—H17B	109.5	C45—C53—H53B	109.5
H17A—C17—H17B	109.5	H53A—C53—H53B	109.5
C9—C17—H17C	109.5	C45—C53—H53C	109.5
H17A—C17—H17C	109.5	H53A—C53—H53C	109.5
H17B—C17—H17C	109.5	H53B—C53—H53C	109.5
C2—C18—H18A	109.5	C38—C54—H54A	109.5
C2—C18—H18B	109.5	C38—C54—H54B	109.5
H18A—C18—H18B	109.5	H54A—C54—H54B	109.5
C2—C18—H18C	109.5	C38—C54—H54C	109.5
H18A—C18—H18C	109.5	H54A—C54—H54C	109.5
H18B—C18—H18C	109.5	H54B—C54—H54C	109.5
C20—C19—C26	125.1 (4)	C56—C55—C62	124.8 (4)
C20—C19—Br2	113.4 (3)	C56—C55—Br4	113.6 (4)
C26—C19—Br2	121.4 (3)	C62—C55—Br4	121.6 (4)
C19—C20—C21	117.6 (4)	C57—C56—C55	117.2 (4)
C19—C20—C36	121.3 (4)	C57—C56—C72	120.3 (5)
C21—C20—C36	121.2 (4)	C55—C56—C72	122.5 (5)
C24—C21—C20	118.4 (4)	C60—C57—C56	119.3 (4)
C24—C21—C22	118.3 (4)	C60—C57—C58	118.7 (5)
C20—C21—C22	123.2 (4)	C56—C57—C58	122.0 (5)
O2—C22—C23	120.6 (5)	O4—C58—C57	122.5 (5)
O2—C22—C21	120.1 (5)	O4—C58—C59	120.3 (5)
C23—C22—C21	119.3 (5)	C57—C58—C59	117.2 (5)
C22—C23—H23A	109.5	C58—C59—H59A	109.5
C22—C23—H23B	109.5	C58—C59—H59B	109.5
H23A—C23—H23B	109.5	H59A—C59—H59B	109.5
C22—C23—H23C	109.5	C58—C59—H59C	109.5
H23A—C23—H23C	109.5	H59A—C59—H59C	109.5
H23B—C23—H23C	109.5	H59B—C59—H59C	109.5
C21—C24—C25	123.9 (4)	C57—C60—C61	124.1 (4)
C21—C24—H24	118.1	C57—C60—H60	117.9
C25—C24—H24	118.1	C61—C60—H60	117.9
C24—C25—C26	119.9 (4)	C60—C61—C62	118.3 (4)
C24—C25—C30	116.6 (4)	C60—C61—C66	117.7 (4)
C26—C25—C30	123.4 (4)	C62—C61—C66	124.0 (4)
C25—C26—C19	114.7 (4)	C61—C62—C55	116.0 (4)
C25—C26—C27	122.5 (4)	C61—C62—C63	121.1 (4)
C19—C26—C27	122.8 (4)	C55—C62—C63	122.9 (4)
C26—C27—C34	110.4 (4)	C64—C63—C71	106.6 (5)
C26—C27—C28	110.1 (4)	C64—C63—C62	110.0 (4)
C34—C27—C28	104.3 (5)	C71—C63—C62	111.1 (4)
C26—C27—C35	111.2 (4)	C64—C63—C70	109.3 (5)
C34—C27—C35	110.1 (4)	C71—C63—C70	110.9 (5)
C28—C27—C35	110.6 (4)	C62—C63—C70	109.0 (4)
C29—C28—C33	111.5 (5)	C65—C64—C63	120.7 (6)
C29—C28—C27	114.0 (5)	C65—C64—C69	115.7 (6)
C33—C28—C27	113.6 (5)	C63—C64—C69	116.5 (6)
C29—C28—H28	105.6	C65—C64—H64	98.9
C33—C28—H28	105.6	C63—C64—H64	98.9
C27—C28—H28	105.6	C69—C64—H64	98.9
C28—C29—C30	116.7 (5)	C64—C65—C66	121.4 (6)
C28—C29—H29A	108.1	C64—C65—H65A	107.0
C30—C29—H29A	108.1	C66—C65—H65A	107.0
C28—C29—H29B	108.1	C64—C65—H65B	107.0
C30—C29—H29B	108.1	C66—C65—H65B	107.0
H29A—C29—H29B	107.3	H65A—C65—H65B	106.7
C31—C30—C32	107.9 (4)	C67—C66—C65	112.1 (5)
C31—C30—C25	111.1 (4)	C67—C66—C61	111.2 (4)
C32—C30—C25	110.6 (4)	C65—C66—C61	109.8 (4)
C31—C30—C29	110.7 (5)	C67—C66—C68	106.6 (5)
C32—C30—C29	107.6 (5)	C65—C66—C68	107.0 (5)
C25—C30—C29	108.9 (4)	C61—C66—C68	110.0 (4)
C30—C31—H31A	109.5	C66—C67—H67A	109.5
C30—C31—H31B	109.5	C66—C67—H67B	109.5
H31A—C31—H31B	109.5	H67A—C67—H67B	109.5
C30—C31—H31C	109.5	C66—C67—H67C	109.5
H31A—C31—H31C	109.5	H67A—C67—H67C	109.5
H31B—C31—H31C	109.5	H67B—C67—H67C	109.5
C30—C32—H32A	109.5	C66—C68—H68A	109.5
C30—C32—H32B	109.5	C66—C68—H68B	109.5
H32A—C32—H32B	109.5	H68A—C68—H68B	109.5
C30—C32—H32C	109.5	C66—C68—H68C	109.5
H32A—C32—H32C	109.5	H68A—C68—H68C	109.5
H32B—C32—H32C	109.5	H68B—C68—H68C	109.5
C28—C33—H33A	109.5	C64—C69—H69A	109.5
C28—C33—H33B	109.5	C64—C69—H69B	109.5
H33A—C33—H33B	109.5	H69A—C69—H69B	109.5
C28—C33—H33C	109.5	C64—C69—H69C	109.5
H33A—C33—H33C	109.5	H69A—C69—H69C	109.5
H33B—C33—H33C	109.5	H69B—C69—H69C	109.5
C27—C34—H34A	109.5	C63—C70—H70A	109.5
C27—C34—H34B	109.5	C63—C70—H70B	109.5
H34A—C34—H34B	109.5	H70A—C70—H70B	109.5
C27—C34—H34C	109.5	C63—C70—H70C	109.5
H34A—C34—H34C	109.5	H70A—C70—H70C	109.5
H34B—C34—H34C	109.5	H70B—C70—H70C	109.5
C27—C35—H35A	109.5	C63—C71—H71A	109.5
C27—C35—H35B	109.5	C63—C71—H71B	109.5
H35A—C35—H35B	109.5	H71A—C71—H71B	109.5
C27—C35—H35C	109.5	C63—C71—H71C	109.5
H35A—C35—H35C	109.5	H71A—C71—H71C	109.5
H35B—C35—H35C	109.5	H71B—C71—H71C	109.5
C20—C36—H36A	109.5	C56—C72—H72A	109.5
C20—C36—H36B	109.5	C56—C72—H72B	109.5
H36A—C36—H36B	109.5	H72A—C72—H72B	109.5
C20—C36—H36C	109.5	C56—C72—H72C	109.5
H36A—C36—H36C	109.5	H72A—C72—H72C	109.5
H36B—C36—H36C	109.5	H72B—C72—H72C	109.5
			
C8—C1—C2—C3	−0.8 (7)	C44—C37—C38—C39	−0.1 (8)
Br1—C1—C2—C3	178.1 (3)	Br3—C37—C38—C39	179.3 (4)
C8—C1—C2—C18	179.8 (4)	C44—C37—C38—C54	178.4 (5)
Br1—C1—C2—C18	−1.3 (6)	Br3—C37—C38—C54	−2.2 (6)
C1—C2—C3—C6	−4.2 (7)	C37—C38—C39—C42	2.7 (7)
C18—C2—C3—C6	175.2 (4)	C54—C38—C39—C42	−175.9 (5)
C1—C2—C3—C4	177.7 (4)	C37—C38—C39—C40	−175.0 (5)
C18—C2—C3—C4	−2.9 (7)	C54—C38—C39—C40	6.5 (8)
C6—C3—C4—O1	137.0 (5)	C42—C39—C40—O3	−126.4 (5)
C2—C3—C4—O1	−44.9 (7)	C38—C39—C40—O3	51.3 (7)
C6—C3—C4—C5	−43.3 (6)	C42—C39—C40—C41	53.5 (6)
C2—C3—C4—C5	134.9 (5)	C38—C39—C40—C41	−128.8 (5)
C2—C3—C6—C7	5.5 (7)	C38—C39—C42—C43	−2.6 (7)
C4—C3—C6—C7	−176.3 (5)	C40—C39—C42—C43	175.2 (4)
C3—C6—C7—C8	−1.4 (7)	C39—C42—C43—C44	−0.3 (7)
C3—C6—C7—C12	179.3 (4)	C39—C42—C43—C48	179.6 (4)
C2—C1—C8—C7	4.6 (7)	C42—C43—C44—C37	2.8 (6)
Br1—C1—C8—C7	−174.2 (3)	C48—C43—C44—C37	−177.2 (4)
C2—C1—C8—C9	−174.9 (4)	C42—C43—C44—C45	−176.2 (4)
Br1—C1—C8—C9	6.3 (6)	C48—C43—C44—C45	3.8 (7)
C6—C7—C8—C1	−3.4 (6)	C38—C37—C44—C43	−2.7 (7)
C12—C7—C8—C1	175.8 (4)	Br3—C37—C44—C43	178.0 (3)
C6—C7—C8—C9	176.1 (4)	C38—C37—C44—C45	176.3 (5)
C12—C7—C8—C9	−4.7 (7)	Br3—C37—C44—C45	−3.0 (6)
C1—C8—C9—C10	168.9 (4)	C43—C44—C45—C52	121.3 (5)
C7—C8—C9—C10	−10.5 (6)	C37—C44—C45—C52	−57.7 (6)
C1—C8—C9—C16	53.7 (6)	C43—C44—C45—C53	−116.2 (5)
C7—C8—C9—C16	−125.8 (4)	C37—C44—C45—C53	64.9 (6)
C1—C8—C9—C17	−68.6 (6)	C43—C44—C45—C46	5.3 (6)
C7—C8—C9—C17	112.0 (5)	C37—C44—C45—C46	−173.6 (5)
C8—C9—C10—C11	42.2 (7)	C52—C45—C46—C47	−151.1 (7)
C16—C9—C10—C11	160.6 (5)	C53—C45—C46—C47	89.9 (7)
C17—C9—C10—C11	−79.9 (6)	C44—C45—C46—C47	−31.9 (8)
C8—C9—C10—C15	172.9 (5)	C52—C45—C46—C51	69.1 (7)
C16—C9—C10—C15	−68.7 (6)	C53—C45—C46—C51	−49.9 (7)
C17—C9—C10—C15	50.8 (6)	C44—C45—C46—C51	−171.7 (5)
C9—C10—C11—C12	−60.2 (7)	C51—C46—C47—C48	−168.0 (6)
C15—C10—C11—C12	168.2 (5)	C45—C46—C47—C48	52.4 (10)
C10—C11—C12—C14	−81.5 (6)	C46—C47—C48—C50	83.7 (8)
C10—C11—C12—C13	159.4 (5)	C46—C47—C48—C43	−39.3 (9)
C10—C11—C12—C7	40.8 (7)	C46—C47—C48—C49	−159.0 (7)
C6—C7—C12—C14	−65.9 (5)	C42—C43—C48—C47	−169.4 (5)
C8—C7—C12—C14	114.9 (5)	C44—C43—C48—C47	10.6 (7)
C6—C7—C12—C13	53.5 (6)	C42—C43—C48—C50	66.4 (6)
C8—C7—C12—C13	−125.7 (5)	C44—C43—C48—C50	−113.6 (5)
C6—C7—C12—C11	170.7 (4)	C42—C43—C48—C49	−51.4 (6)
C8—C7—C12—C11	−8.5 (6)	C44—C43—C48—C49	128.6 (5)
C26—C19—C20—C21	−0.7 (7)	C62—C55—C56—C57	3.3 (7)
Br2—C19—C20—C21	178.6 (3)	Br4—C55—C56—C57	−174.7 (3)
C26—C19—C20—C36	−178.3 (4)	C62—C55—C56—C72	−178.4 (5)
Br2—C19—C20—C36	1.0 (6)	Br4—C55—C56—C72	3.6 (6)
C19—C20—C21—C24	−4.1 (7)	C55—C56—C57—C60	1.0 (7)
C36—C20—C21—C24	173.5 (4)	C72—C56—C57—C60	−177.3 (4)
C19—C20—C21—C22	173.9 (4)	C55—C56—C57—C58	−178.4 (5)
C36—C20—C21—C22	−8.4 (7)	C72—C56—C57—C58	3.2 (7)
C24—C21—C22—O2	136.4 (5)	C60—C57—C58—O4	−125.9 (6)
C20—C21—C22—O2	−41.6 (7)	C56—C57—C58—O4	53.5 (8)
C24—C21—C22—C23	−43.7 (6)	C60—C57—C58—C59	54.4 (6)
C20—C21—C22—C23	138.2 (5)	C56—C57—C58—C59	−126.1 (5)
C20—C21—C24—C25	4.4 (7)	C56—C57—C60—C61	−3.8 (7)
C22—C21—C24—C25	−173.7 (5)	C58—C57—C60—C61	175.6 (4)
C21—C24—C25—C26	0.3 (7)	C57—C60—C61—C62	2.3 (7)
C21—C24—C25—C30	−177.8 (4)	C57—C60—C61—C66	−178.6 (4)
C24—C25—C26—C19	−4.8 (6)	C60—C61—C62—C55	1.8 (6)
C30—C25—C26—C19	173.2 (4)	C66—C61—C62—C55	−177.3 (4)
C24—C25—C26—C27	173.7 (4)	C60—C61—C62—C63	−176.5 (4)
C30—C25—C26—C27	−8.3 (7)	C66—C61—C62—C63	4.4 (7)
C20—C19—C26—C25	5.1 (7)	C56—C55—C62—C61	−4.7 (7)
Br2—C19—C26—C25	−174.1 (3)	Br4—C55—C62—C61	173.2 (3)
C20—C19—C26—C27	−173.4 (4)	C56—C55—C62—C63	173.6 (5)
Br2—C19—C26—C27	7.4 (6)	Br4—C55—C62—C63	−8.5 (6)
C25—C26—C27—C34	−122.2 (5)	C61—C62—C63—C64	7.3 (7)
C19—C26—C27—C34	56.2 (6)	C55—C62—C63—C64	−171.0 (5)
C25—C26—C27—C28	−7.7 (6)	C61—C62—C63—C71	125.0 (5)
C19—C26—C27—C28	170.8 (5)	C55—C62—C63—C71	−53.2 (6)
C25—C26—C27—C35	115.2 (5)	C61—C62—C63—C70	−112.5 (5)
C19—C26—C27—C35	−66.4 (6)	C55—C62—C63—C70	69.2 (6)
C26—C27—C28—C29	40.8 (7)	C71—C63—C64—C65	−153.1 (8)
C34—C27—C28—C29	159.2 (5)	C62—C63—C64—C65	−32.6 (11)
C35—C27—C28—C29	−82.4 (6)	C70—C63—C64—C65	87.0 (9)
C26—C27—C28—C33	170.1 (5)	C71—C63—C64—C69	57.5 (9)
C34—C27—C28—C33	−71.5 (6)	C62—C63—C64—C69	178.0 (7)
C35—C27—C28—C33	46.9 (7)	C70—C63—C64—C69	−62.3 (9)
C33—C28—C29—C30	168.5 (5)	C63—C64—C65—C66	46.8 (13)
C27—C28—C29—C30	−61.3 (7)	C69—C64—C65—C66	−163.6 (7)
C24—C25—C30—C31	−66.6 (6)	C64—C65—C66—C67	94.1 (9)
C26—C25—C30—C31	115.3 (5)	C64—C65—C66—C61	−30.0 (11)
C24—C25—C30—C32	53.1 (6)	C64—C65—C66—C68	−149.4 (9)
C26—C25—C30—C32	−125.0 (5)	C60—C61—C66—C67	60.7 (6)
C24—C25—C30—C29	171.2 (5)	C62—C61—C66—C67	−120.2 (5)
C26—C25—C30—C29	−6.9 (7)	C60—C61—C66—C65	−174.7 (5)
C28—C29—C30—C31	−80.7 (6)	C62—C61—C66—C65	4.4 (7)
C28—C29—C30—C32	161.6 (5)	C60—C61—C66—C68	−57.2 (6)
C28—C29—C30—C25	41.7 (7)	C62—C61—C66—C68	121.9 (5)
